# Estimation of change limits (deltacheck) in clinical laboratory

**DOI:** 10.1515/almed-2020-0114

**Published:** 2021-04-01

**Authors:** Maria-José Castro-Castro, Lourdes Sánchez-Navarro

**Affiliations:** Clinical Laboratory, Bellvitge University Hospital, Barcelona, Catalonia, Spain

**Keywords:** autoverification, change limits, deltacheck, plausibility control, validation

## Abstract

**Objectives:**

Change limits, more commonly called delta check, are those in which a change in a patient’s measured result in relation to their corresponding preceding measurement is suspected of being erroneous and should be considered as a doubtful result. The aim of this study was to provide change limits for some biochemical and haematological quantities to detect doubtful measured results and to assess its effectiveness to detect erroneous results for their application in and the standardization of the plausibility control.

**Methods:**

Change limits have been estimated for 13 biochemical and 6 haematological quantities. For each quantity, relative differences (*D*), expressed as a percentage between the two consecutive measured results from the same patient (from scheduled laboratory requests), were calculated. From these differences (*D*), the *p*5 and *p*95 percentiles of the data distribution were calculated. To assess the effectiveness of the change limits to detect laboratory errors, 43 erroneous laboratory reports, containing different biochemical and haematological quantities, were obtained from the standard laboratory plausibility control procedure.

**Results:**

From the 43 erroneous laboratory reports, 31 (72%) were due to endovenous administration errors and 12 (28%) were due to mislabeling errors. All erroneous laboratory reports were detected when the change limits of the quantities were combined and applied together.

**Conclusions:**

The best combination of quantities, which detect all the erroneous reports in the same specimen were: potassium, albumin, creatinine, glucose and haemoglobin.

## Introduction

Plausibility control, commonly called “autoverification”, is defined as the set of procedures used to determine whether a patient’s measured result is valid according to previously-established clinical and biological criteria [1], [2], [3], [4], [5]. This is the last process in the postanalytical phase and is carried out to assure the quality of a clinical laboratory report before it is released to the requester. This procedure is also a useful tool for detecting doubtful measured results, which can be erroneous due to sampling errors, typing errors, interferences etc. [6], [7], [8], [9], [10]. Measured results that are deemed satisfactory in this process are plausible and acceptable and can be delivered to the requester in the clinical laboratory report.

Plausibility control includes some tools that can be used to detect doubtful measured results, including alert limits (a range of values that, when exceeded, consider a measured result as doubtful) [11], change limits (a range of values of the difference between two consecutive measured results observed in the same patient that, when exceeded, consider a measured result as doubtful) [11] and prediction limits (a range of values of a biological quantity which are established from another physiopathologically-related biological quantity that, when exceed, consider a measured result as doubtful) [12]. If stablished thresholds values are exceeded, some workflow process have to be done to determine if the measured result is real or an error. These actions include to check information about the specimen, about the diagnosis, repeating measurement, requesting a new sample… [13].

Change limits, more commonly called delta check, are those in which a change in a patient’s measured result in relation to their corresponding preceding measurement is suspected of being erroneous and should be considered as a doubtful result. Under physiological conditions, the observed difference between two measured results of the same biological quantity in the same individual is the combined effect of premetrological, metrological and biological intraindividual variability. This difference will vary depending on the circumstances, but in a way that is generally considered a ‘reasonable’ variation (i.e. the measured result agrees with the previous result). When a measured result does not agree with the previous result, it is considered as a doubtful measured result. Several approaches have been used to establish change limits, including empiric data, based on experience of the laboratory staff, clinicians or selected from the literature [14], [15]. Change limits can also be obtained from objective data using data based on intraindividual (within-subject) biological variability [16], [17], or the use of percentiles of the population distribution of the differences [11, 17–20]. The time interval over which change is measured is another important point to take into account for the establishment of thresholds values. Most of the published studies show a high degree of variation of the selected time interval, probably due to lack of specific recommendations and the absence of scientific evidences. Nevertheless, time intervals are usually short, between three and seven days for chemistry and hematology quantities [13].

Plausibility control can be applied in a nonstandardised or standardised manner. Nonstandardised plausibility control is performed by laboratory specialists who perform a visual inspection of the measured results. This process has a high degree of subjectivity and interindividual variation, as the specialists apply their own rules with a lack of standardisation in the algorithms used or the criteria applied. Conversely, computerisation of plausibility control allows standardisation of the process. This kind of plausibility control employs rules, algorithms and sophisticated systems to detect measured results that exceed the alert limits, the change limits (delta check) or prediction limits. It automatically delivers measured results that are considered acceptable to the requester [1]. Computerisation has several advantages, including the elimination of interindividual variation, improvement of the process efficiency and the saving of time and effort.

Although partially-computerised plausibility control systems are available in most laboratory information systems, plausibility control is still performed in a nonstandardised way in many clinical laboratories. Autoverification systems are used primarily in big-size clinical laboratories with high workloads. Nevertheless, only a part of laboratory which employs autoverification systems, between 25 and 40%, depending on the literature use delta check as a way to detect potential erroneous reports [21], [22]. The main reasons are diverse: the study of delta check and the verification of its effectiveness is expensive and labor-intensive, so publications that provide verified change limits to configure in the laboratory information systems are limited. On the other hand, all equations to assess delta checks are not available in all laboratory information systems. The aim of this study was to provide change limits for some biochemical and haematological quantities to detect doubtful measured results and to assess its effectiveness to detect erroneous results for their application in and the standardization of the plausibility control.

## Materials and methods

The study was performed at the Clinical Laboratory of Bellvitge University Hospital (Barcelona, Spain) which is a 900-bed teaching hospital specialized in adult patient care. Bellvitge University Hospital has all the medical-surgical specialties, except pediatrics and obstetrics. It admits about 35,000 patients each year, of which about 28.5% of its registrations are from high complexity (oncology, cardiology, nephrology, gastroenterology, …) and also performs about 12,000 major surgeries and about 180 transplants each year. The clinical laboratory is accredited according to the National Standard ISO 15189.

In order to estimate change limits, measured results of scheduled laboratory requests were taken from the database maintained in the clinical laboratory using the Omnium information system (Roche Diagnostics). Results from scheduled biochemical quantities, measured using the Cobas c701 analyser (Roche Diagnostics), and results from scheduled hematological quantities, measured using the Sysmex XN analyser (Kobe, Japan), from inpatients were obtained from 01 January 2018 to 31 December 2018.

This long period of time has been chosen to obtain the greatest number of inpatients with previous results.

The selected quantities were based on its effectiveness to detect mislabeled errors and contamination-related errors, following the CLSI guideline: “Use of delta checks in the medical laboratory” [21].

The selected biochemical quantities were serum concentrations of albumin, alanine aminotransferase, alkaline phosphatase, aspartate aminotransferase, bilirubin, calcium (II), chloride, creatinine, gamma-glutamyltransferase, glucose, potassium ion, sodium ion and urea.

The selected haematological quantities were: number concentration of erythrocytes, leukocytes and thrombocytes; mass concentration of haemoglobin; volume fraction (hematocrit) and entitic volume of erythrocytes (MCV).

To estimate change limits, results below the corresponding limit of detection were considered to be equal to the numerical value of that limit. Time interval within which two sequential results of a patient will be evaluated to calculate delta check limits was one year. The 90th percentile of the days between two results for the same patient was calculated.

For each quantity, relative differences (D), expressed as a percentage between the two consecutive measured results from the same patient, were calculated using the equation below, taking into account the most recent result (current result) and the previous result:

$$D=\frac{\text{Current result}-\text{Previous result}}{\text{Previous result}}\times 100\left(\%\right)$$

A negative value indicates that there was a decrease in the current measurement compared to the previous measurement, and a positive value indicates that there was an increase in the current measurement compared to the previous measurement of the same quantity in the same patient.

From these differences (*D*), the *p*5 and *p*95 percentiles of the data distribution were calculated. Therefore this excluded 10% of the results, with *p*5 being the lower change limit and *p*95 the upper change limit.

To assess the effectiveness of the change limits to detect laboratory errors, 43 erroneous laboratory reports, containing different biochemical and haematological quantities, were selected from the register of issues of the clinical laboratory reported between January and April 2018. When some error reports due to contamination for endovenous administration or mislabeling errors were suspected, a second sample were requested. To consider an error was based in of the final and subjective decision of the laboratory staff in applying the standard plausibility control procedure, after comparing the results of the first and the second samples.

The erroneous reports contained two types of errors: contamination for endovenous administration errors (type 1 laboratory error) and mislabeling errors (type 2 laboratory error). For each quantity and laboratory report, the calculated change limits were applied. The effectiveness to detect erroneous laboratory reports was calculated in a single way for each quantity and also in a general way when the all change limits were applied together. The best combination of quantities was assessed. In order to achieve this, the quantities with the best effectiveness to detect errors were successively added until the greatest number of erroneous reports was achieved.

SPSS v.17 was used (SPSS, Chicago, US) for all statistical analysis.

## Results

Table 1 shows the change limits of the scheduled biochemical quantities and Table 2 shows the change limits of the scheduled hematological quantities. Both tables describe the quantities according to the International Union of Pure and Applied Chemistry (IUPAC) and the International Federation of Clinical Chemistry and Laboratory Medicine (IFCC) recommended syntax [23], and express them using the International System of Units. These tables show the time interval between two results for the same patient (median with interquartilic range), the number of measured results for each quantity (n), the number and fraction (%) of measured results that had a previous result in the same patient, and the change limits (%). The median of days between two results for the same patient was three days (with an interquartilic range between four and five days) and the 90th percentile was lower than 18 days.

**Table 1:** Change limits from the biochemical scheduled quantities.

Biochemical quantity, units	n	Time interval median (IQR)	n with previous results, %	Lower *p*5 change limit, %	Upper *p*95 change limit, %
S; Alanine aminotransferase; cat.c., µkat/L	39,558	3 (4)	23,677 (60%)	−57	109
S; Albumin; mass c., mg/L	34,715	3 (4)	19,564 (56%)	−17	21
S; Alkaline phosphatase; cat.c., µkat/L	32,660	3 (5)	18,449 (56%)	−39	38
S; Aspartate aminotransferase; cat.c., µkat/L	29,388	3 (5)	15,294(52%)	−54	144
S; Bilirubin; subst. c., µmol/L	29,830	3 (4)	15,571 (52%)	−46	101
S; Calcium (II); subst. c., mmol/L	18,088	3 (5)	8,243 (46%)	−10	10
S; Creatinine; subst. c., µmol/L	42,385	3 (4)	26,570 (63%)	−25	40
S; Glucose; c.sust, mmol/L	41,656	3 (4)	25,841 (62%)	−37	68
S; γ-Glutamyltransferase; cat.c., µkat/L	37,424	3 (4)	21,920 (59%)	−58	62
S; Potassium ion; subst. c., mmol/L	39,944	3 (4)	21,887 (55%)	−19	22
S; Sodium; subst. c., mmol/L	42,162	3 (4)	26,309 (62%)	−4	4
S; Urea; subst. c., mmol/L	39,289	3 (4)	24,521 (62%)	−44	77

Time interval: median (IQR) = time interval within which two sequential results of a patient has been evaluated to calculate delta check limits, expressed like median an interquertilic range (IQR).

**Table 2:** Change limits from haematological scheduled quantities.

Hematological quantity	n	Time interval median (IQR)	n with previous results, %	Lower *p*5 change limit, %	Upper *p*95 change limit, %
B; Erythrocytes; num.c., 10^12^/L	40,256	3 (4)	23,890 (59%)	−18	20
B; Haemoglobin; mass c., g/L	40,447	3 (4)	24,632 (61%)	−18	20
B; Erythrocytes; vol.fr., %	40,447	3 (4)	24,632 (61%)	−18	20
B; Erythrocytes; entitic vol., fL	40,449	3 (4)	24,636 (61%)	−4	4
B; Leukocytes; num. c., 10^9^/L	39,938	3 (4)	23,614 (59%)	−43	77
B; Platelets; num c., 10^9^/L	40,159	3 (4)	24,056 (60%)	−43	60

Time interval: median (IQR) = time interval within which two sequential results of a patient has been evaluated to calculate delta check limits, expressed like median an interquertilic range (IQR).

From the 43 erroneous laboratory reports, 31 (72%) were due to endovenous administration errors and 12 (28%) were due to mislabeling errors.

Table 3 shows the number and fraction of total erroneous reports detected for each quantity and for each type of error.

**Table 3:** Percentage of errors detected.

Quantity, units	n	Errors detected, n (%)	Type 1 errors, n	Type 1 errors detected, n (%)	Type 2 errors, n	Type 2 errors detected, n (%)
S; Potassium ion; subst. c., mmol/L	42	27 (64)	30	21 (70)	12	6 (50)
S; Albumin; mass c., mg/L	23	14 (61)	18	9 (50)	5	5 (100)
S; Creatinine; subst. c., µmol/L	43	25 (58)	31	15 (48)	12	10 (83)
S; Glucose; c.sust, mmol/L	43	24 (56)	31	21 (68)	12	3 (25)
B; Haemoglobin; mass c., g/L	42	23 (55)	30	17 (57)	12	6 (50)
B; Erythrocytes; num.c., 10^12^/L	42	23 (55)	30	17 (57)	12	6 (50)
S; Sodium; subst. c., mmol/L	43	22 (51)	31	17 (55)	12	5 (42)
B; Erythrocytes; vol.fr., %	42	21 (50)	30	15 (50)	12	6 (50)
S; Urea; subst. c., mmol/L S; γ-	32	14 (44)	24	8 (33)	8	6 (75)
S; Bilirubin; subst. c., µmol/L	28	11 (39)	22	7 (32)	6	4 (67)
S; Calcium (II); subst. c., mmol/L	19	7 (37)	14	5 (36)	5	2 (40)
B; Leukocytes; num. c., 10^9^/L	42	15 (36)	30	10 (33)	12	5 (42)
S; Alkaline phosphatase; cat.c., µkat/L	25	6 (24)	19	5 (26)	6	1 (17)
B; Erythrocytes; entitic vol. (MCV), fL	42	10 (24)	30	6 (20)	12	4 (33)
S; Alanine aminotransferase; cat.c., µkat/L	36	8 (22)	26	4 (15)	10	4 (40)
S; Aspartate aminotransferase; cat.c., µkat/L	27	4 (15)	21	3 (14)	6	1 (17)
B; Platelets; num c., 10^9^/L	42	6 (14)	30	3 (10)	12	3 (25)
S; Glutamyltransferase; cat.c., µkat/L	33	4 (12)	25	4 (16)	8	0 (0)

Type 1 errors = endovenous administration errors; type 2 errors = mislabeling errors.

All erroneous laboratory reports were detected when the change limits of the quantities were combined and applied together.

**Figure 1:**
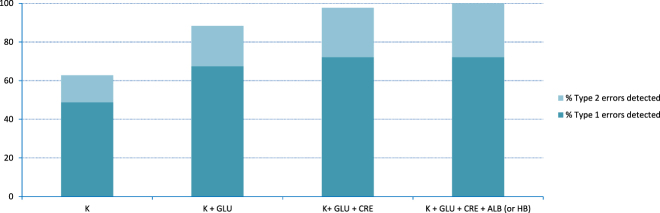
Percentage of errors detected by different combinations of quantities. K, serum concentration of potassium ion; GLU, serum concentration of glucose; CRE, serum concentration of creatinine; ALB, serum concentration of albumin; HB, mass concentration of haemoglobin. Type 1 errors: endovenous administration errors; type 2 errors: mislabeling errors.

The combination of five quantities with the highest percentages of error detection was: concentration of potassium ion, creatinine, albumin, glucose and haemoglobin. Figure 1 shows the percentage of errors detected by different combinations of quantities.

## Discussion

In plausibility control, the change limit is the most important tool for detecting most clinical laboratory errors (i.e. contamination of the samples with intravenous administration or identification of sample errors).

Some points have to be into consideration to establish its own change limits in a clinical laboratory: selecting analytes which change limits are useful, origin of the change limits (percentiles of differences distributions, reference change value …), change limits calculation mode (absolute differences, relative differences …), selecting the partitioning criteria based on the origin of the population (inpatients, outpatients …), the time intervals between specimens and evaluating the effectiveness of the estimated limits.

The quantities with low individual variability are preferred to select as the entitic volume of erytrocytes (MCV). An index of individuality <0.60 suggests that the quantity is useful for specimen misidentification. In this study, the selected quantities were based in the CLSI document “Use of delta checks in the medical laboratory” [21]. In this guideline, criteria are established to choose the quantities that can be most useful for detecting the most frequent errors in the clinical laboratory. These criteria are based on intra and inter-individual biological variability, and index of individuality. Measurands that have a high individuality are likely to perform well when used for detecting misidentified specimens [24].

Several approaches to setting change values can be used, based on the purpose of its use in the laboratory. The selected model based on the percentiles of the distribution allows data from the clinical laboratory to be used, therefore the estimated change limits include the variability of the metrological variation (imprecision and bias of the measuring systems) and the biological and the pathological variability based on the population characteristics (inpatient or outpatient) [11]. This model is classified like the change limit derived from the distribution of delta values in the patient population, which was cited in the Clinical and Laboratory Standards Institute document [21].

Concerning the calculation mode, the selected model to estimate change limits has to be related to how they can be applied in the laboratory information system. In this laboratory, Infinity (Roche Diagnostics) was the implemented laboratory information system, and the change limits could be set in percentage or absolute values.

How these differences are estimated may alter between different laboratory information systems, with change limits estimated using other equations being nonuseful. In a previous publication, an equation which accounted for the highest and lowest result between two consecutive results in the same patient was used [11]:

$$D=\frac{\text{Highest result}-\text{Lowest result}}{\text{Lowest result}}\times 100$$

This current study was conducted to adapt the equations for estimating the change limits and to show the development of this model. Change limits were estimated for a large number of biochemical quantities, and it is important to emphasise that these limits differ depending on the origin of the sample (scheduled or stat laboratory). In general, change limits from a stat laboratory should be wider than those from a scheduled laboratory.

Tables 1 and 2 shows that biochemical and haematological quantities with a narrow reference interval and narrow homeostatic body control have lower change limits; for example, the change limits were lower than ±10% for sodium, calcium (II) and volume of erythrocytes. The values obtained for each quantity are in general higher than most publications. Two recent papers [25], [26] reveal the following change: 3.2 and 4.4 for sodium ion, 6.2 and 7.8 for calcium, 9.7 and 11.5 for albumin, 18.3 and 20.8 for creatinine, 18.8 and 23 for alkaline phosphatase, as well as 16 and 16.7% for glucose. These limits are derived from the biological variation that only includes physiological variability. Change limits based on percentiles also include pathological and iatrogenic variability which is expected to be higher. They are particularly relevant when plausibility control is applied to inpatients. This fact is especially important in the case of glucose, in which its concentration is directly related with a very usual procedure as an intravenous administration. In this way, change limits based on percentiles will avoid an unmanageable number of doubtful clinical laboratory reports that finally they will be issued after a detailed inspection (false positive).

Concerning the time intervals to establish change limits, the 90% of change limits were obtained with a time interval less than 18 days. Despite there no clear recommendations, these time intervals are quite short, according to some publications.

The change limits detailed in this study would identify 10% of the doubtful results detected for each quantity. This percentage was chosen arbitrarily and by taking into account the availability of professional staff to review doubtful results for the laboratory. The selection of the appropriate percentile depends on the necessities of each clinical laboratory and its effectiveness to detect specimen errors. There are few publications about the validation or the verification of the effectiveness of the change limits. Most of them are based on simulations of errors, in which clinical laboratory intentionally make the specimens mislabeled, contamined or otherwise compromised. In our study the laboratory errors were real, based in the final decision of laboratory staff applying the current plausibility control process. The calculated change limits had to be at least as good as the current plausibility control system was.

The types of errors that are most frequently observed in the clinical laboratory (endovenous administration and mislabeling errors) have been studied. Regarding other types of errors, nothing has been mentioned, since they are detected by other tools. Hemolyzed samples are detected by measuring the sample’s hemolysis index, and a comment of “Hemolyzed sample” is reported in the quantities without providing the result. Coagulated samples are detected by visual inspection of the samples or by obtaining results that exceed the applied alert limits. Other types of errors, such as interference due to drug administration, are less frequent in the laboratory. Despite all this, it may be interesting for the future to develop another study aimed at the detection of these kinds of errors.

Table 3 and Figure 1 show that the best quantities to detect laboratory errors were: potassium, albumin, creatinin, glucose, haemoglobin, erythrocytes and sodium. For each kind of laboratory error, the best quantities to detect contamination from endovenous administration were: potassium, creatinine, glucose, haemoglobin, erythrocytes, sodium and haematrocrit. The best quantities to detect mislabeling samples were: albumin, creatinine, urea and bilirubin. There was no quantity that has the ability to detect all the erroneous laboratory reports when applied individually. In the case of potassium concentration, despite being the best quantity to detect erroneous laboratory reports, can only detect around 64% of them. As well known, a laboratory report is made up of a set of quantities, and the joint application of change limits to different quantities increases its effectiveness to detect laboratory errors. Figure 1 shows that all erroneous reports can be detected with the combination of few quantities which are quite usually requested in most laboratory tests. The best combination of quantities were: potassium, albumin, creatinine, glucose and haemoglobin. Nevertheless, to achieve this, all of these quantities would be requested at the same time.

Change limits not always can be applied because there is not previous result or the time interval between consecutive results exceeds the stablished limit. In our study, taking into account that the exported data corresponded to inpatients, only a 46–63% had a previous result in the time interval studied (stat requests from the same patient have not been taken into account). If change limits were stablish in outpatients, the proportion of the presence of a previous result of the same patient, it is sure to be lower and this must be taken into account when implementing change limits. In these cases, other plausibility tools, as alert limits o prediction limits, play a decisive rule to detect doubtful results. On the other hand, the quantities requested by the clinician not always are the same so the effectiveness of the change limits is directly related to those requested quantities.

The results from this study reveal an approximation to implement change limits in a clinical laboratory and show that each laboratory should estimate its own limits and to be configured in a laboratory information system. This study also offers the possibility to adopt these effective change limits. For these purpose, these limits should previously be verified according to the patient population.

Regarding the elapsed time to calculate and apply the deltachecks, there is no evidence of what time is better to apply to use these limits and there are not any recommendation to this [27], [28].

Lee et al. showed that change limits based on percentiles provide symmetrical distribution for some of the quantities (i.e. sodium, potassium, protein and albumin) obtained in this study [22]. A recent publication showed that a higher number of analytes used for change limits would increase the sensitivity of detecting problems; however, the number of false alerts may also be higher using this method [29].

The limitation of this study was that false positive rate to detect erroneous laboratory reports were no assessed. Change limits were only applied to erroneous laboratory reports. To decide if a laboratory report is wrong or not, it is necessary to carry out a set of actions that may generate patient discomfort, as obtaining a new sample. Nevertheless, the limits were stablished taking into account the availability of professional staff to review doubtful results for the laboratory. By the other hand, it would also be interesting to carry out this study combining the results from stat as well as scheduled requests.

## Conclusions

This study shows the change limits obtained in a clinical laboratory with a high volume of samples in which the computerisation and standardisation of plausibility control are essential. With this tool, clinical laboratories may standardise the plausibility control process and minimise the intraindividual variability due to subjectivity. In the same way, the time invested and effort caused by the manual review of all doubtful measured results would be decreased.

## References

[1] Fuentes Arderiu X, Basart Arraut M, Bosch Ferrer A, Castiñeiras Lacambra M, López Martínez R, Miro Balagué J (2008). Proposed guidelines for the final review of measurement results in the clinical laboratory. Accred Qual Assur.

[2] Fuentes X, Ferrer ÀB (2008). Guia per a la revisió final dels resultats de mesura en el laboratori clínic. Assoc Catalana Ciències Lab Clínic Vitr Verit.

[3] Harm K, Wegener M, Dieckvoß E, Nage l H, Voigt K (1982). Palusibility control in clinical chemistry results. Fresenius’ Z für Anal Chem.

[4] Büttner J, Stamm D (1995). Plausibilitätskontrolle. Ärztliche Verwndung von Klin Befunden Greiling H, Gressner AM (Hrsg) Lehrb der Klin Chemie und Pathobiochemie 3 Aufl.

[5] Fuentes Arderiu X, Castro Castro MJ, Sánchez Navarro L, Dot Bach D (2013). Validació i control de la plausibilitat dels resultats. Vitr Verit.

[6] Plebani M (2010). The detection and prevention of errors in laboratory medicine. Ann Clin Biochem.

[7] Plebani M, Carraro P (1997). Mistakes in a stat laboratory: types and frequency. Clin Chem.

[8] Carraro P, Plebani M (2007). Errors in a stat laboratory: types and frequencies 10 years later. Clin Chem.

[9] Plebani M (2006). Errors in clinical laboratories or errors in laboratory medicine?. Clin Chem Lab Med.

[10] García Santamarina S, Cocchiararo A, Fuentes Arderiu X (2006). Transcriptions and ISO 15189. Clin Chem Lab Med.

[11] Castro Castro MJ, Dot Bach D, Candás Estébanez B, Cano Corres R, Fuentes Arderiu X (2011). Estimation of alert and change limits and its application in the plausibility control. Accred Qual Assur.

[12] Castro Castro MJ, Candás Estébanez B, Solé Enrech G, Fuentes Arderiu X (2009). Use of prediction equations for reviewing measurement results in the clinical laboratory. Accred Qual Assur.

[13] Young DS, Harris EK, Cotlove E (1971). Biological and analytic components of variation in long-term studies of serum constituents in normal subjects. IV. Results of a study designed to eliminate long-term analytic deviations. Clin Chem.

[14] Ladenson JH (1975). Patients as their own controls: use of the computer to identify “laboratory error”. Clin Chem.

[15] Lacher DA (1990). Relationship between delta checks for selected chemistry tests. Clin Chem.

[16] Young DS, Harris EK, Cotlove E (1971). Biological and analytic components of variation in long-term studies of serum constituents in normal subjects. IV. Results of a study designed to eliminate long-term analytic deviations. Clin Chem.

[17] Lacher DA (1990). Relationship between delta checks for selected chemistry tests. Clin Chem.

[18] Wheeler LA, Sheiner LB (1977). Delta check tables for the technicon analyzer. Clin Chem.

[19] Lacher DA, Connelly DP (1988). Rate and delta checks compared for selected chemistry tests. Clin Chem.

[20] Sheiner LB, Wheeler LA, Moore JK (1979). The performance of delta check methods. Clin Chem.

[21] Clinical and Laboratory Standards Institute (2016). Use of delta checks in the medical laboratory – EP33.

[22] Lee J, Kim S, Kwon HJ, Lee HK, Kim Y, Kim Y (2016). Clinica Chimica Acta usefulness of biological variation in the establishment of delta check limits. Clin Chim Acta.

[23] IUPAC-IFCC (1995). Properties and units in the clinical laboratory sciences. Syntax and semantic rules. Pure &App/Chem.

[24] Schifman RB, Talbert M, Souers RJ (2017). Delta check practices and outcomes. Arch Pathol Lab Med.

[25] Ko DH, Il PH, Hyun J, Kim HS, Park MJ, Shin DH (2017). Utility of reference change values for delta check limits. Am J Clin Pathol.

[26] Hong J, Cho EJ, Kim HK, Lee W, Chun S, Min WK (2020). Application and optimization of reference change values for delta checks in clinical laboratory. J Clin Lab Anal.

[27] Randell EW, Yenice S (2019). Delta checks in the clinical laboratory. Crit Rev Clin Lab Sci.

[28] Ma C, Cheng X, Xue F, Li X, Yin Y, Wu J (2020). Validation of an approach using only patient big data from clinical laboratories to establish reference intervals for thyroid hormones based on data mining. Clin Biochem.

[29] Ovens K, Naugles C (2012). How useful are delta checks in the 21st century: a stochastic-dynamic model of specimen mix-up and detection. J Pathol Inf.

